# Pectus excavatum: the effect of tricuspid valve compression on cardiac function

**DOI:** 10.1007/s00247-024-05971-z

**Published:** 2024-07-09

**Authors:** Molly K. Carroll, Adam W. Powell, William D. Hardie, Karla E. Foster, Bin Zhang, Victor F. Garcia, Vinicius P. Vieira Alves, Rebeccah L. Brown, Robert J. Fleck

**Affiliations:** 1 University of Cincinnati College of Medicine, Cincinnati, OH USA; 2 Department of Pediatrics, University of Cincinnati College of Medicine, Cincinnati, OH USA; 3 Division of Pulmonary Medicine, Cincinnati Children’s Hospital Medical Center, Cincinnati, OH USA; 4 Department of Radiology, Cincinnati Children’s Hospital Medical Center, 3333 Burnet Avenue, MLC 5031, Cincinnati, 45229 OH USA; 5 The Heart Institute, Cincinnati Children’s Hospital Medical Center, Cincinnati, OH USA; 6 Department of Radiology, University of Cincinnati College of Medicine, Cincinnati, OH USA; 7 Division of Biostatistics and Epidemiology, Cincinnati Children’s Hospital Medical Center, Cincinnati, OH USA; 8 Division of Pediatric Surgery, Cincinnati Children’s Hospital Medical Center, Cincinnati, OH USA

**Keywords:** Cardiopulmonary function, Haller index, Depression index, Sternal tilt, Correction index, Cardiac compression index, Chest wall, Pectus excavatum

## Abstract

**Background:**

Pectus excavatum (PE) is a common congenital chest wall deformity with various associated health concerns, including psychosocial impacts, academic challenges, and potential cardiopulmonary effects.

**Objective:**

This study aimed to investigate the cardiac consequences of right atrioventricular groove compression in PE using cardiac magnetic resonance imaging.

**Materials and methods:**

A retrospective analysis was conducted on 661 patients with PE referred for evaluation. Patients were categorized into three groups based on the degree of right atrioventricular groove compression (no compression (NC), partial compression (PC), and complete compression(CC)). Chest wall indices were measured: pectus index (PI), depression index (DI), correction index (CI), and sternal torsion.

**Results:**

The study revealed significant differences in chest wall indices between the groups: PE, NC=4.15 ± 0.94, PC=4.93 ± 1.24, and CC=7.2 ± 4.01 (*P*<0.0001). Left ventricle ejection fraction (LVEF) showed no significant differences: LVEF, NC=58.72% ± 3.94, PC=58.49% ± 4.02, and CC=57.95% ± 3.92 (*P*=0.0984). Right ventricular ejection fraction (RVEF) demonstrated significant differences: RVEF, NC=55.2% ± 5.3, PC=53.8% ± 4.4, and CC=53.1% ± 4.8 (*P*≥0.0001). Notably, the tricuspid valve (TV) measurement on the four-chamber view decreased in patients with greater compression: NC=29.52 ± 4.6; PC=28.26 ± 4.8; and CC=24.74 ± 5.73 (*P*<0.0001).

**Conclusion:**

This study provides valuable insights into the cardiac consequences of right atrioventricular groove compression in PE and lends further evidence of mild cardiac changes due to PE.

**Graphical abstract:**

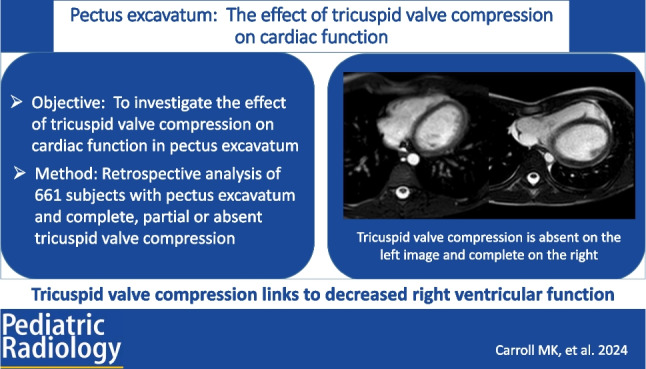

**Supplementary Information:**

The online version contains supplementary material available at 10.1007/s00247-024-05971-z.

## Introduction

Pectus excavatum (PE) is common with an incidence of 1 in 300 to 400, and a male to female ratio of 2:1 [1]. The ensuing chest wall deformity can impact patients’ psychosocial, academic [2], and cardiopulmonary function and be associated with chest pain, palpitations, and decreased exercise tolerance [3–6]. The cardiopulmonary effects of PE can range from very mild to severe and appear to be related to the severity of the chest wall deformity; however, the cardiopulmonary effect does not have a tight relationship to the severity of deformity [3]. One could state that the pathophysiologic effects of PE are controversial, but most surgeons treating this condition would state that the pathophysiologic effects of PE are real [6–8]. Given that the changes affecting the heart function usually do not have a clinical manifestation, the controversy continues. Medical evidence is mixed on the impact PE has on cardiopulmonary function, but many of the cardiopulmonary exercise tests, pulmonary function tests, and cardiac functional evaluations are still within normal limits [3, 4, 9]. As a population, patients with PE have cardiac systolic function on the low side of average with the RVEF and LVEF bell curves of the PE group shifted leftward from the normal bell curve [3, 6].

Cardiopulmonary signs and symptoms were recognized in case reports and small series of case in the early, pioneering days of pectus repair and is nicely summarized in a paper by Wachtel et al. [6]. In their report of 13 subjects, they reviewed the literature from 1939 to 1956. The degree of PE was severe in most cases and symptoms were extensive. Yet, in most cases, the subject had substantial or complete resolution of symptoms post-surgery. Of course, these early studies selected for only the severe subjects. In 1974, Guller and Hable looked at 54 subjects with moderate or severe PE and state that on physical examination had finding suggestive of cardiovascular disease: murmurs on auscultation, electrocardiogram changes, vector electrocardiogram changes, and chest X-ray changes [7]. Nearly all were asymptomatic, 52 out of 54, which led them to conclude these findings were due to displacement of the heart and had minimal clinical significance [7]. In the modern era of cardiac MRI, Saleh et al. compare pectus excavatum with match normal controls using CMR and found that the RVEF was reduce by 6% in the pectus group compared to the controls but did not find a change in pulmonary circulation time or perfusion indices [10]. Another group evaluated the cardiac systolic function pre- and postoperatively showing that RVEF was reduced preoperatively as a group and postoperatively either remained stable or improved. RVEF less than 45% predicted improvement [11]. Using CMR and stress echocardiography, PE subjects were found to have lower peak exercise capacity, changes in both the left and right ventricular E/A ratio, increased tricuspid gradient, and abnormal septal motion with flattening during inspiration [12]. This brief review shows that PE affects cardiac systolic function, particularly affecting the right side of the heart.

The evolution of PE repair has evolved from an uncommonly performed, truly invasive surgery to a minimally invasive surgery which is performed in centers throughout the world. In the early days, resection of the anterior chest wall was practiced from 1910 to 1920. The Ravitch procedure was developed in 1949 which left many children with constrictive chest wall physiology leading to a “modified Ravitch procedure” using Adkins struts that dominated from the mid-1950s until the 1980s. Nuss introduced the first “Nuss bar” repair in 1987 [13, 14]. Since the first “Nuss bar” repair, the technique and equipment have been refined, creating the minimally invasive repair of PE that is used today [14]. Whereas earlier methods of repair were relatively risky and more painful, after the advent of the minimally invasive repair of pectus excavatum, repair of PE has become increasingly common.

Around the same time that Nuss was developing his novel technique for repair, J. Alex Haller et al. published an article that employed CT to evaluate chest wall shape of patients with PE and introduced a CT-based pectus index (PI) [15]. A PI of >3.25 defined a cutoff value for a group of patients that underwent repair in their institution [15]. This was adopted as the threshold for performance of PE repair. Subsequently, there has been a proliferation of cross-sectional imaging indices from CT and MRI.

The chest wall deformity of PE causes variable compression of the right ventricle which is associated with decreased right ventricle function. Tandon et al. found that compression of the right ventricle free wall resulted in significantly lower right ventricle ejection fraction when compared to those without compression, and further decrease with compression at the right atrioventricular groove [16]. The hypothesis of this study is that compression of the atrioventricular groove results in a progressive decrease in right ventricular systolic function. This study aims to fully assess the effect that right atrioventricular groove compression has on the cardiac systolic function as measured by cardiac MRI and relate this to standard measures of pectus excavatum.

## Patients and methods

Approval was obtained from the institutional review board to retrospectively examine the electronic medical record of patients with PE referred to our chest wall center for evaluation. Demographic data included age, gender, race, weight, and height. Inclusion criteria were referral to the chest wall center and cardiac MRI. Exclusion criteria included congenital heart disease, prior chest wall surgery, underlying pulmonary disease, and connective tissue disorders. If a patient had repeat studies, only the most recent presurgical study was included. All patients seen by our chest wall center undergo cardiac MRI unless there is a contraindication.

Cardiac magnetic resonance imaging was performed on an Ingenia 1.5-Tesla scanner (Philips Healthcare, Best, Netherlands) to assess pectus indices, cardiac compression, and cardiac function. The protocol included axial single-shot steady-state free precession (SSFP) with cardiac end systolic gating and respiratory gating, standard cine SSFP with retrospective electrocardiogram gating obtained during suspended expiratory breath hold. These were obtained in the vertical long axis, horizontal long axis (four-chamber view), short-axis, and left ventricular outflow tract planes. Our standard protocol also included cine steady-state free precession in a trans axial plane, free breathing cine phase contrast of the aortic valve, main pulmonary artery, and the atrioventricular valves on one phase contrast cine slice. At the end, a 3D SSFP, cardiac, and respiratory-gated whole-heart sequence was performed. No contrast was administered for the exam.

The cardiac functional values for the right and left ventricles were obtained from the clinical database. Therefore, they were performed by multiple cardiac-trained radiologists and cardiologists. The radiologist and cardiologist both review the images on each exam and produce a single report. The cardiac functional values collected were indexed to the body surface area, and included right ventricular ejection fraction (RVEF), end diastolic volume (RVEDV_i_), end systolic volume (RVESV_i_), stroke volume (RVSV_i_), and end diastolic mass (RVEDM_i_). Our institution adopted definitions for all cardiac MRI cutoff values based on the literature which provides clinical consistency. Low normal is defined as RVEF of ≥48 to <50% and mild right ventricular functional impairment is defined as ≥40 to <48%. The analogous variables were collected for the left ventricle: ejection fraction (LVEF), end diastolic volume (LVEDV_i_), end systolic volume (LVESV_i_), stroke volume (LVSV_i_), and end diastolic mass (LVEDM_i_). All variables are indexed to the body surface area. Our institutional cutoff values for the LV are as follows: low normal is defined as LVEF of ≥52 to <55% and mild right ventricular functional impairment is defined as ≥40 to <52%.

The chest wall indices were collected from the clinical reports. The following indices are included: pectus (Haller) index (PI); correction index (CI) [1]; depression index [17] (DI); chest wall asymmetry ratio [18]; and sternal torsion [19]. Normal average and standard deviation for PI reported by Haller et al. in the original paper was 2.56 ± 0.35 [15], and >3.25 is considered the threshold for PE repair. A CI threshold of 10.0% and above is proposed to define pectus patients with significant defects who should be considered for repair [18]. The depression index (DI), a measure of the pectus depth divided by the width of a vertebral body, has a reported threshold for DI to indicate a PE deformity severe enough to consider surgical correction is >0.2 [17]. Cardiac compression indices included modified cardiac compression index (mCCI) and modified cardiac asymmetry index (mCAI). These are labeled “modified” because they were modified for measurement on our MRI images which are obtained during end expiration and not during full inspiration on CT as described by Kim et al. [18] who used the xiphoid as a point of reference. We have modified the mCCI and mCAI so that it is measured at a uniform anatomic level on the heart since cardiac MRI is performed in expiratory breath hold. We measure at the level one slice above the edge of the visible eustachian valve on the axial single shot SSFP MR images, which approximates the level of the mid-tricuspid valve (TV) on axial images. Kim et al. report normal values of 1.1 to 2.0 for the CCI and 0.9 to 1.4 for the CAI [18]. A guide to performing these measurements is provided in the Supplemental Material found online. These measurements were also the result of clinical studies and were performed by the radiologists and cardiologists who both read each exam at our institution.

**Fig. 1:**
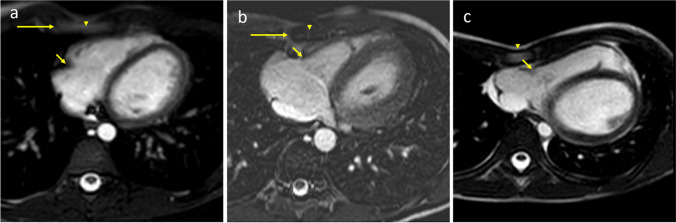
Axial single-shot steady-state free precession with cardiac end systolic gating and respiratory gating (**a**, **b**, **c**). 16-year-old male with pectus index of 3.9 and tricuspid valve (TV) compression absent (**a**). The TV (short arrow) is to the right of the sternal edge (long arrow) and not in contact with the sternum. Arrowhead is the center of the sternum. 11-year-old female with pectus index of 3.6 and partial TV compression (**b**). The tricuspid valve (short arrow) is to the left of the sternal edge (long arrow) in contact with the sternum but not beyond the center of the sternum (arrowhead). During diastole, the tricuspid valve is still in contact with the lung pleura (Supplemental Fig. 1). 14-year-old male with pectus index of 6.3 and complete TV compression (**c**). The tricuspid valve (short arrow) is to the left of the middle of the sternum (arrowhead) and in contact with the sternum. During diastole, the tricuspid valve is in contact with the chest wall (Supplemental Fig. 2)

Additionally, novel measurements of the cardiac structures include a semi-quantitative assessment of TV compression (Fig. 1). TV compression was evaluated as absent if the TV remained to the right of the sternum on the axial single-shot SSFP (Fig. 1a) which is obtained at end systole. TV compression was evaluated as partial compression if the valve plane was in contact with sternum, but to the right of midline (Fig. 1b, Supplemental Fig. S1) and complete if the valve was to the left of sternal midline (Fig. 1c, Supplemental Fig. S2). Categorization of TV compression was performed by cardiac imaging attending and second year medical student with, respectively, 20 years and 8 weeks of experience with cardiac imaging and performing these measurements.

**Fig. 2:**
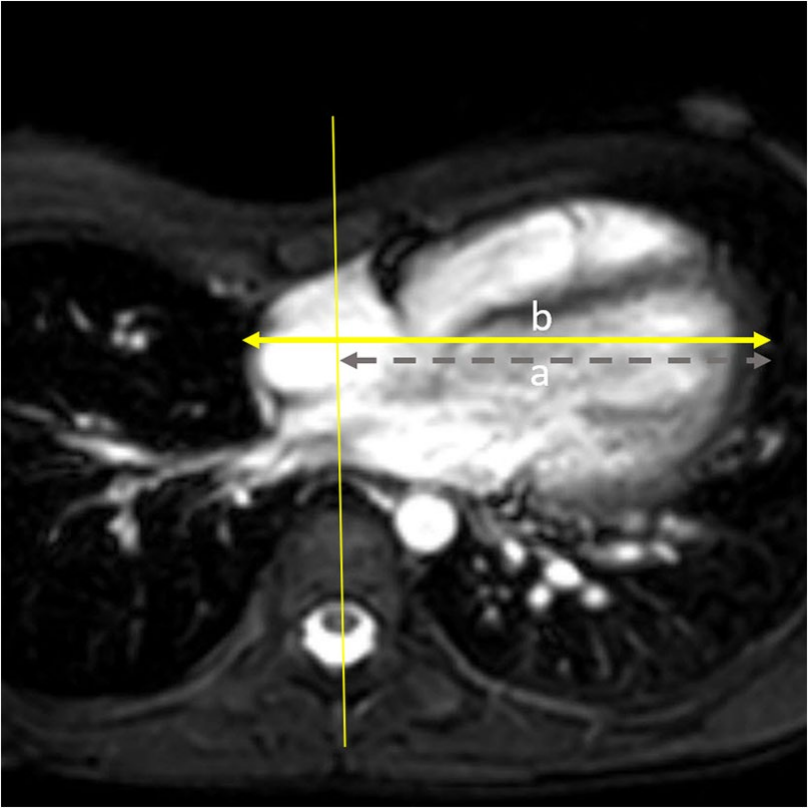
Percentage of the cardiac transverse diameter displaced left of midline (cardiac displacement). Axial single-shot steady-state free precession of a 13-year-old male with pectus index of 7.1. The thin yellow line is the center of the chest, and the gray dashed line (a) represents the portion of the heart left of midline. The yellow double arrow line (b) is the transverse diameter of the heart. Percent of cardiac displacement=(a/b)×100

**Fig. 3:**
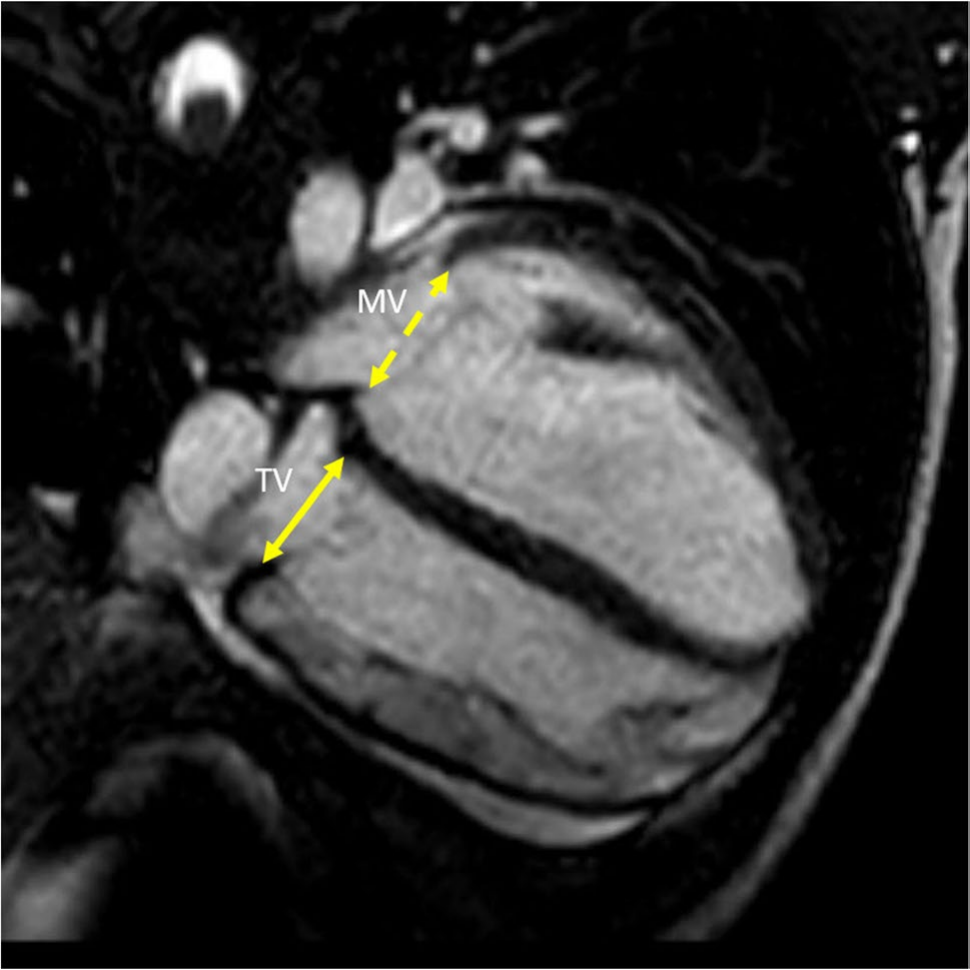
Tricuspid (TV) and mitral valve (MV) measurement. Four-chamber steady-state free precession cine of a 13-year-old male with pectus index of 7.1. The yellow double arrow line labeled TV is the tricuspid valve measurement. The yellow dash double arrow line (MV) is mitral valve measurement

**Fig. 4:**
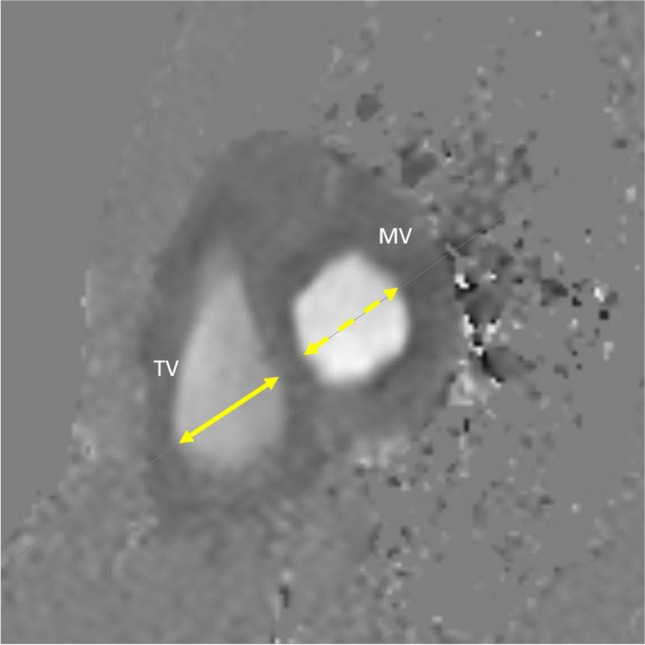
Tricuspid (TV) and mitral valve (MV) measurement. Cine phase contrast across the atrioventricular valve during peak flow. 15-year-old male with pectus index of 4.5. The yellow double arrow line (TV) is the tricuspid valve measurement. The yellow dash double arrow line (MV) is mitral valve measurement

**Fig. 5:**
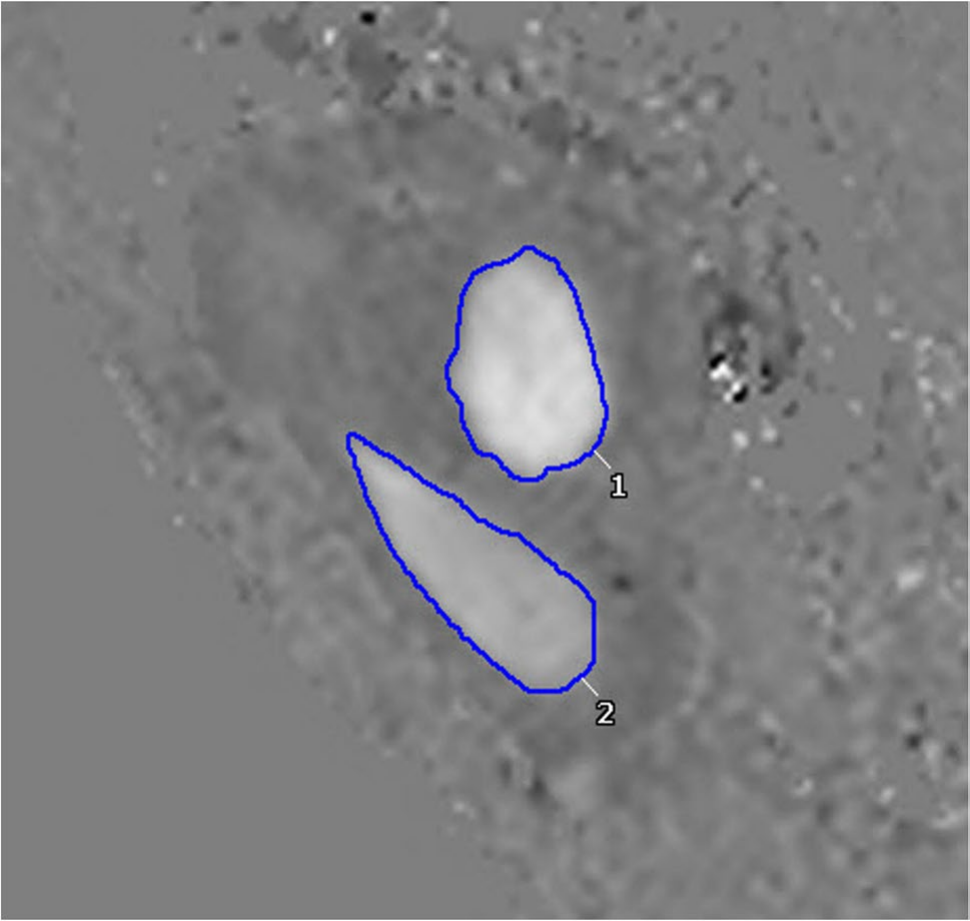
Tricuspid (TV) and mitral valve (MV) area measurement. Cine phase contrast across the atrioventricular valves during peak flow. 14-year-old male with pectus excavatum and pectus index of 6.3. TV compression was scored as complete in this patient. The contour labeled 1 represents the MV area of 6.66 cm^3^ and the contour labeled 2 is the TV with an area of 6.79 cm^3^

The percentage of the cardiac transverse diameter displaced left of midline was also determined (Fig. 2). The TV and mitral valve (MV) were measured on the four-chamber cine images during diastole and the ratio of the two calculated as TV diameter divided by mitral valve diameter (Fig. 3). The TV and MV were also measured on cine phase contrast imaging (Fig. 4). The valves were measured with a linear measurement that would reflect the valve measurement on the four-chamber cine, but measured during peak flow across the valve which would represent early diastole. Additionally, the cross-sectional area of the flow across valve planes was measured (Fig. 5). Phase contrast cine MRI was performed according to accepted standards. A group of 100 measurements were repeated to obtain the interobserver agreement. The categorical values for classifying TV compression were scored by second year medical student (8 weeks of training and experience) and post medical school clinical research fellow with 2 years of experience. The research fellow utilized descriptions of the measures similar to that contained in this paper. The Kappa statistic for classifying TV compression was determined using the cardiothoracic attending with 20 years of experience as the reference.

### Statistical analysis

Descriptive analysis was performed to describe the characteristics of the variables of interest. Continuous variables were presented as mean and standard deviation (SD) and were compared between three groups using one-way ANOVA. The interobserver agreement was evaluated using intraclass correlation coefficient with 95% confidence interval. All analyses were performed using SAS version 9.4 (SAS Institute Inc., Cary, NC, USA). A *P*-value less than 0.05 was considered statistically significant.

## Results

A total of 661 different patients were identified with a complete set of data, duplicate exams on patients were removed, and all had pectus excavatum. The median age of these patients was 15 (13, 16) and 532 (80%) were male. The overall median pectus index was 4.5 (3.9, 5.6). TV compression was present in 195 (29.5%), partial TV compression was present in 139 (21.0%), and no TV compression was present in 327 (49.5%) patients. The average age of the patients with TV compression was 16.3 ± 4.6 years, partial compression 15.3 ± 3.1 years, and those without TV compression were 14.9 ± 3 years (*P*=0.0006). There were no differences in height, weight, body mass index, or body surface area (Table 1).

**Table 1:** Demographics of each group. *BMI* body mass index, *BSA* body surface area

	No *N*=327	Partial *N*=139	Complete *N*=195	*P*-value
Age (years)	14.95 ± 3.05	15.26 ± 3.07	16.39 ± 4.57	0.0006
Height (cm)	170.86 ± 10.29	171.19 ± 9.51	172.18 ± 11.54	0.38
Weight (kg)	56.92 ± 12.11	55.31 ± 10.69	57.14 ± 12.96	0.33
BMI	19.31 ± 2.76	18.73 ± 2.45	19.09 ± 3.09	0.12
BSA (m^2^)	1.66 ± 0.22	1.63 ± 0.2	1.68 ± 0.23	0.16

### Pectus-related indices

Of the chest wall indexes that are routinely reported at our institution, there were highly significant differences between the subjects with complete TV compression and those without or with partial TV compression (Table 2).

**Table 2:** Chest wall and cardiac compression indices results

	No *N*=327	Partial *N*=139	Complete *N*=195	*P*-value
Haller index (HI)	4.15 ± 0.94	4.93 ± 1.24	7.2 ± 4.01	<0.0001
Depression index (DI)	0.47 ± 0.22	0.56 ± 0.25	0.82 ± 0.3	<0.0001
Correction index (CI) (%)	23.77 ± 11.08	30.7 ± 12.14	44.76 ± 15.53	<0.0001
Cardiac compression index (CCI)	2.28 ± 0.53	2.83 ± 0.73	3.64 ± 1.47	<0.0001
Cardiac asymmetry index (CAI)	1.36 ± 0.27	1.64 ± 0.37	2.21 ± 0.87	<0.0001
Chest wall asymmetry index (CWAI)	0.98 ± 0.11	1.01 ± 0.08	1.08 ± 0.64	0.0005
Sternal torsion (degrees)	10.73 ± 8.39	11.73 ± 7.89	18.5 ± 10.68	<0.0001

The pectus index (PI) measured as follows: without compression, 4.15 ± 0.94; with partial compression, 4.93 ± 1.24; and complete TV compression, 7.2 ± 4.01 (*P<*0.0001). The depression index (DI), a measure of the pectus depth divided by the width of a vertebral body [17], showed a similar pattern of mild worsening between no compression and partial compression, and a substantial increase in subjects with complete TV compression (Fig. 1). Subjects with no compression had a DI of 0.47 ± 0.2; subjects with partial compression had a DI of 0.56 ± 0.2; and subjects with complete TV compression demonstrated a DI of 0.82 ± 0.3 (*P*<0.0001).

Again, a similar pattern was observed in the correction index (CI): no compression, 23.8% ± 11.1; partial compression, 30.7% ± 12.1; and subject with complete TV compression demonstrated a CI of 44.8% ± 15.5 (*P*<0.0001). Two other abnormal chest wall indexed values that showed the same consistent pattern were the chest wall asymmetry index (CWAI) and sternal torsion: chest wall asymmetry index (CWAI), no compression=0.98 ± 0.11, partial compression=1.01 ± 0.08, and complete TV compression=1.08 ± 0.64, *P*=0.0005; and sternal torsion (degrees), no compression=10.7 ± 8.3, partial compression=11.7 ± 7.9, and complete TV compression=18.5 ± 10.7 (*P*<0.0001).

Cardiac compression indices, when evaluating the mCCI and mCAI, continue to parallel the chest wall indices pattern. The mCCI measured as follows: no compression=2.3 ± 0.5; partial compression=2.8 ± 0.7; and complete TV compression=3.6 ± 1.5, *P*<0.0001. The mCAI measured as follows: no compression=1.4 ± 0.3; partial compression=1.6 ± 0.4; and complete TV compression=2.2 ± 0.9 (*P*<0.0001).

### Cardiac indices

Cardiac functional measurements were all normalized by dividing the individual patient values by body surface area. It is a notable observation that there was no statistical difference in the height, weight, body mass index, or body surface area between groups (Table 1). The main finding in cardiac functional measurements is that there are differences in the ejection fraction and end systolic volume index of the right ventricle between patients with and without sternal compression of the TV (Table 3).

**Table 3:** Cardiac function indices results. *RV* right ventricle, *LV* left ventricle, *EF *ejection fraction, *EDV* end diastolic volume, *BSA* body surface area, *ESV* end systolic volume, *ED* end diastolic, *ml* milliliter, *m*^*2*^ meter squared, *gm* grams

	No *N*=327	Partial *N*=139	Complete *N*=195	*P*-value
RV EF (47–67)	55.17 ± 5.33	53.76 ± 4.36	53.07 ± 4.81	<0.0001
RV EDV/BSA (ml/m^2^) (74–134)	94.91 ± 15.24	97.05 ± 15.59	98.17 ± 17.31	0.07
RV ESV/BSA (ml/m^2^) (26–62)	42.68 ± 9.58	45.14 ± 9.78	46.47 ± 11.28	0.0001
RV stroke volume/BSA (ml/m^2^)	52.18 ± 7.4	51.83 ± 9.5	51.59 ± 8.3	0.72
RV ED mass/BSA (gram/m^2^)	18.75 ± 5.36	19 ± 4.88	19.86 ± 6.39	0.09
LV EF (57–77)	58.72 ± 3.94	58.49 ± 4.02	57.95 ± 3.92	0.1
LV EDV/BSA (ml/m^2^) (68–112)	89.25 ± 13.35	89.69 ± 13.16	90.81 ± 14.33	0.44
LV ESV/BSA (ml/m^2^) (16–44)	37.03 ± 7.35	37.35 ± 7.45	38.49 ± 8.15	0.1
LV stroke volume/BSA (ml/m^2^)	52.12 ± 7.86	52.52 ± 8.21	52.41 ± 7.65	0.85
LV ED mass/BSA (gm/m^2^) (47–87)	46.14 ± 10.13	45.22 ± 10.1	44.88 ± 9.76	0.34

### Right ventricle

The RVEF measured as follows: no compression=55.2% ± 5.3; partial compression=53.8% ± 4.4; and complete TV compression=53.1% ± 4.8, *P*<0.0001. RVEDV_i_ (ml/m^2^) showed a non-statistically significant increasing trend (no compression=94.9 ± 9.6; partial compression=97.1 ± 15.6; and complete TV compression=98.2 ± 17.3, *P*=0.07), whereas RVESV_i_ (ml/m^2^) demonstrated statistically significant differences (no compression=42.7 ± 9.6; partial compression=45.1 ± 9.8; and complete TV compression=46.7 ± 11.3, *P*<0.0001). RVSV_i_ (ml/m^2^) was maintained across groups: no compression=52.2 ± 7.4; partial compression=51.9 ± 9.5; and complete TV compression=51.6 ± 8.3, *P* = 0.7. RVEDM_i_ (g/m^2^) differences were statistically insignificant: no compression=18.8 ± 5.4; partial compression=19 ± 4.9; and complete TV compression=19.9 ± 6.4, *P*=0.09 (Table 3).

### Left ventricle

The left ventricle did not demonstrate differences in the cardiac indices: LVEF, no compression=58.72% ± 3.94, partial compression=58.49% ± 4.02, and complete TV compression=57.95% ± 3.92, *P*=0.1. LVEDV_i_ (ml/m^2^) differences were not statistically significant: no compression=89.25 ± 13.35; partial compression = 89.69 ± 13.16; and complete TV valve compression=90.81 ± 14.33, *P*=0.44. LVESV_i_ (ml/m^2^) was not significantly different between categories: no compression=37.03 ± 7.35; partial compression=37.35 ± 7.45; and complete TV compression=38.49 ± 8.15, *P*=0.1. LVSV_i_ (ml/m2) was maintained across groups: no compression=52.12 ± 7.86; partial compression=52.52 ± 8.21; complete compression=52.41 ± 7.65, *P*=0.85. LVEDM_i_ (g/m^2^) differences were statistically insignificant: no compression=46.14 ± 10.13; partial compression=45.22 ± 10.1; and complete TV compression=44.88 ± 9.76, *P*=0.34. The majority of the LVEF were within normal limits, but each group had several subjects with mildly low LVEF. In the group with no compression = 17 (5.2%) had an EF < 48%; partial compression = 4 (2.9%), and complete TV compression = 17 (8.7%).

### Valve measurements and heart displacement

The novel measurements of TV and MV diameter during diastole and their ratio were statistically significant to a high degree (Table 4). The measurements of the TV on the four-chamber view (mm) are as follows: no compression=29.52 ± 4.6; partial compression=28.26 ± 4.8; and complete compression=24.74 ± 5.73 (*P*<0.0001). The measurement of the MV on the four-chamber view (mm) was less significant and increased with each degree of chest wall compression: no compression=31.03 ± 4.6; partial compression=31.8 ± 4.49; and complete compression=32.26 ± 4.79, *P*=0.01. The ratio of the TV divided by the MV on the four-chamber view is as follows: no compression=0.97 ± 0.18; partial compression=0.9 ± 0.16; and complete compression=0.78 ± 0.2 (*P*<0.0001). The width and cross-sectional area of the TV and MV as measured on phase contrast MRI were also measured. The TV measurement (mm) is as follows: no compression=30.08 ± 4.33; partial compression=28.08 ± 5.34; and complete TV compression=24.34 ± 5.87 (*P*<0.0001). The cross-sectional area of the TV decrease was highly significant: no compression=10.25 ± 2.26; partial compression=9.8 ± 2.8; and complete TV compression=8.35 ± 2.48 (*P*<0.0001). A less significant but similar pattern was observed for the MV with a linear measurement (mm) but not with cross-sectional area measurement (cm^2^) with *P*=0.03 and *P*=0.06.

**Table 4:** Atrioventricular valve measurements and heart displacement results. *TV* tricuspid valve, *MV *mitral valve, *4ch* four-chamber, *mm* millimeter, *cm*^*2*^ centimeter

	No *N*=327	Partial *N*=139	Yes *N*=195	*P*-value
4ch TV (mm)	29.52±4.6	28.26 ± 4.8	24.74 ± 5.73	<0.0001
4ch MV (mm)	31.03 ± 4.6	31.8 ± 4.49	32.26 ± 4.79	0.01
Ratio of 4ch TV/4ch MV	0.97 ± 0.18	0.90 ± 0.16	0.78 ± 0.2	<0.0001
Percentage displaced	0.65 ± 0.05	0.7 ± 0.05	0.79 ± 0.07	<0.0001
Width of TV (mm)	30.08 ± 4.33	28.08 ± 5.34	24.34 ± 5.87	<0.0001
Width of MV (mm)	31.58 ± 3.91	32.22 ± 3.87	30.67 ± 3.94	0.04
Area TV (cm^2^)	10.25 ± 2.26	9.8 ± 2.8	8.35 ± 2.48	<0.0001
Area MV (cm^2^)	7.32 ± 1.65	7.63 ± 1.8	7.02 ± 1.44	0.07

### Interobserver agreement

An interobserver agreement was determined between the two junior readers, a second year medical student with 8 weeks of experience and training with feedback and a physician research fellow with 2 years of experience with instructions as outlined in the manuscript. The intraclass correlation coefficient (Table 5) was excellent (>0.90) for heart transverse and heart left of midline measurements and good (>0.70) for measurements of the four-chamber TV, linear width and area of the TV, and area of the MV on the cine phase contrast images. However, the interclass correlation was poor between the linear measurements of the MV on the four-chamber cine and the cine phase contrast images.

**Table 5:** Interobserver agreement. *TV* tricuspid valve, *MV* mitral valve, *4ch* four-chamber, *mm* millimeter, *cm*^*2*^ centimeter

	ICC	95% CI	*P* value
4ch TV (mm)	0.79	0.72–0.85	<0.01
4ch MV (mm)	0.44	0.29–0.56	<0.01
Heart Transverse (mm)	0.91	0.87–0.93	<0.01
Heart left of midline (mm)	0.90	0.86–0.93	<0.01
Percentage displaced	0.82	0.76–0.87	<0.01
Width of TV (mm)	0.77	0.69–0.83	<0.01
Width of MV (mm)	0.21	0.05–0.36	0.02
Area TV (cm^2^)	0.75	0.67–0.82	<0.01
Area MV (cm^2^)	0.72	0.63–0.79	<0.01

The agreement between the evaluations of TV compression was measured using the Kappa statistic (Table 6). Second year medical student with 8 weeks of training and instruction had a Kappa=0.72, whereas research fellow using instructions as outlined in this paper demonstrated a Kappa=0.63. Both values are substantial in agreement with the standard reference, a cardiovascular imaging attending with 20 years of experience.

**Table 6:** Agreement between reader and the reference. *CI* confidence interval

	Kappa	95% CI	*P*-value
Medical student	0.72	(0.62, 0.81)	<0.01
Research fellow	0.63	(0.52, 0.74)	<0.01

## Discussion

This study demonstrates that increasing compression or restriction of the tricuspid valve results in lower RV systolic function compared to the group without compression of the tricuspid valve. For the tricuspid valve to be compressed by the sternum, a displacement of the heart toward the left hemithorax is required and is evident in the percentage that the heart is displaced into the left thorax across midline. Not unsurprisingly, all these are related to increased chest wall deformity indices. Overall, the relationship of chest wall indexes to decreased RV systolic function as a cause and effect is elusive and certainly is not a direct one-to-one relationship. Below we discuss the findings and possible suggestions as to the cause of decreased RV systolic function in the low normal range.

The demographics of the three groups of patients (no compression, partial compression, and complete compression of the TV) were notably consistent in height, weight, body mass index, and body surface area, and only different in age with patients progressively older with increasing severity of TV compression. The consistency of the demographics is likely due to the limited range of age in this pediatric referral center. The age difference is probably because the severity of the chest wall deformity worsens with age and the worsening of the chest wall deformity causes a greater shift in the cardiac structures to the left [20].

The finding of a consistent progressive worsening of the chest wall indices from no compression to complete TV compression is not surprising. First, the chest wall indices are all tied together by the shape of the chest wall with the use of either the distance from the sternum to spine, or the depth of the chest wall deformity in the numerator and another measure of body size in the denominator. The exceptions to this calculation are the CWAI and the sternal torsion which were both statistically significant. Both are tied to the chest wall deformity, though. Sternal torsion was measured in degrees, but the direction of the rotation was not noted as clockwise or counterclockwise which may come into play with shifting of the heart leftward. Ours was only an absolute value. Capunay et al. evaluated the effect of sternal torsion on PE and found that HI, CI, CCI, and TV/MV width were all affected by sternal torsion and that if the right side was down (counterclockwise), the effect was greatest [19]. If counterclockwise was recorded as negative and clockwise as positive, the results may be different because of the competing positive and negative values in the analysis. Here, as an absolute value in degrees, it may only be linked to the severity of the chest wall deformity.

The mCCI and mCAI also reflect the severity of chest wall deformity and the lack of space for the heart in its usual location. As such, it is not surprising that the percentage of the heart displacement to the left of midline is also increased. Compression of the TV depends on the displacement of the heart to the left by the chest wall deformity. The severity of the chest wall deformity is related to cardiac function in many studies of PE [3–5].

However, the main hypothesis of this study was that increased compression of the right atrioventricular valve leads to decreased right ventricular systolic function. The ejection fraction of the right ventricle demonstrated a statistically significant difference with the highest difference occurring when progressing from no TV compression to partial TV compression, a difference of 1.41% and 0.69% to complete TV compression. It would seem the critical change is when the chest wall deformity is even partially limiting the motion of the TV and base of the RV. In patients without compression of the TV, the excursion of the TV annulus is sliding against the curvature of the pericardium that is up against lung pleura, whereas when partial or complete TV compression is present, the excursion is sliding horizontally up against the sternum and chest wall, limiting motion to two dimensions. This compression also limits the expansion of the base of the RV. The RVEDV_i_ trends toward significance and RVESV_i_ demonstrated a statistically significant increase. The overall RVSV is unchanged. This suggests that there is mild dilation of the RV to maintain stroke volume. The lack of statistical significance for the RVEDV_i_ may be due to several factors. First, the RV is known to be more difficult to contour consistently due to the trabeculations, partial volume effects due to RV shape, and difficulty contouring at the plane of the atrioventricular valve resulting in higher interobserver and intraobserver variability than the left ventricle [21–24]. Second, our research uses the clinical data derived by many readers rather than having a small group repeat the contours. The percent displaced, that portion of the heart displaced to the left of midline, is also increased from 65% ± 5 when no compression of the TV is present to 79% ± 7 when there is complete TV valve compression. When the heart is more displaced into the left chest, the RV is more able to dilate because of a larger interface with the lung, the rotation of the heart posteriorly and the anterior chest wall outward. This is supported by the abnormal RV systolic and diastolic function found during stress echocardiography and decreased tricuspid valve area compared to children without PE [9]. Perhaps, using newly available FDA approved, clinically utilized artificial intelligence contour detection, and review of contours by one or two expert scorers would provide cleaner and more consistent results that are statistically significant [22, 25, 26].

We looked at the TV size in relation to the degree of categorical compression: no TV compression, partial TV compression, and complete TV compression. In each group, the measurement of the TV decreased. When evaluating this on the four-chamber cine during diastole, the TV size (mm) decreased with each group from no compression to complete compression. The largest decrease is from partial compression to complete compression. This was also true when the TV was indexed to the size of the MV. Initially, the thought was that the MV would reflect the patient size, but the MV became larger with progressive chest wall deformity and compression of the TV. This is an interesting observation that is not due to the size of the subjects. Age may be a possibility in that each TV compression group is older as one progressed from no TV compression to complete TV compression. However, since there is no size difference in height, weight, body mass index, or body surface area, it suggests another possibility. Leftward displacement of the heart pushes the MV further away from the spine and “freeing it up” may allow the MV to expand slightly when it is not compressed. Another issue with measuring the TV on the four-chamber cine is that the TV flow is teardrop shaped and the width measured depends on where the placement of the imaging plane is and where it bisects the TV. It is for this reason that we also measure the TV linearly and in cross-sectional area on the phase contrast images. These were measured at peak flow because it is easiest to visualize the valve flow edges than the valve edges, whereas the four-chamber view was measured during diastole. On the PC of the TV, the relationship between TV compression was the same for both the linear and cross-sectional area measurements. Therefore, it is conclusive that TV compression is present when the sternum/chest wall deformity and TV are in contact throughout the cardiac cycle. Interestingly, Capunay et al. performed a similar evaluation when looking at sternal torsion and reported similar findings [19]. The change in the cross-sectional area of the TV is unlikely to cause a substantial change in resistance to flow across the valve but adds support to the change in the shape of the right atrium and right atrioventricular junction.

Hypotheses for the cause of changes in right heart function are many, but in this study, the small change in the TV size raises the question as to whether that is contributing to the cause. We do not think the change in the TV size has any significant contribution to the decreased RV ejection fraction. From principles in fluid mechanics, the small change in size would cause no significant resistance or flow difference. However, the decrease in the TV valve size is not only associated with more compression of the heart in general but also with more deformity of the atrium and the right ventricle base. We hypothesize that one of the factors related to decreased RVEF in PE is the effect of the shape change of the atrium, TV, and RV on the normal flow vortices that maintain the momentum of blood in a normal heart as it enters the right atrium from the inferior and superior vena cava and crosses the TV [27, 28]. Prior authors have expressed several other hypothetical causes including twisting of the venous inflow, cardiac compression of the atria, limitation of cardiac expansion, and respiratory limitation that contributes to the mild cardiac function changes in PE [18]. It would be very interesting to evaluate these vortices in normal subjects versus patients with PE using 4D flow imaging. Others have found RV diastolic function and systolic function in PE worsened with exercise stress [9]. The mid-RV circumferential strain was found to be decreased by Mazur et al. with a concomitant increase in basal and apical strain which was hypothesized to be compensatory [29]. Since a large fraction of patients with PE have compression of the mid-RV wall, this compression could restrict the end diastolic expansion of the RV circumference resulting in decreased strain and contractility. As the compression of the heart moves toward the AV groove, the affected volume of RV would have larger decreased strain and thus decrease function. Evaluation of strain based on where the sternum is compressing the RV is feasible.

The left ventricular cardiac indices did not show statistical significance, but it is interesting that LVEF trended downward with LVESV_i_ and approached statistical significance. The differences were very small, and the LVEDV_i_ showed no such trend and stroke volume index were very consistent between the subjects. Overall, the cardiac function in PE patient is shifted to the low part of the normal cardiac functional distribution [3, 9, 12, 30].

There are limitations to this study. First, the values used for cardiac function and chest wall indices were derived from clinical reports which likely increased variability over having a few experts reproduce these analyses. Second, it is a retrospective study without normal controls. Third, some would state that using body surface area to normalize the cardiac functional values is overly influenced by obesity. However, patients with PE tend to be a very asthenic group and only four had a BMI over 30. Fourth, tricuspid valve compression related to Haller index, depression index, shift of the heart to the left, and other measures of chest wall deformity suggest that the usefulness of this finding is limited. Additionally, the small decrease in the right ventricular systolic function is of doubtful clinical significance as this study does not include clinical symptomology or outcomes. Overall, it does describe changes in the region of the tricuspid valve and adds to the cardiac function in pectus excavatum literature.

## Conclusion

Our study demonstrates that compression of the tricuspid valve is associated with decreased function of the right ventricle and increased pectus excavatum indices. In most patients, RV systolic function remains in the normal range but on the left side of the bell curve. It also provides further evidence of decreased cardiac function due to PE highlighting the need for further research to better understand cause and effect between chest wall deformity and cardiac function in affected individuals.

### Electronic supplementary material

Below is the link to the electronic supplementary material.

Supplementary Material 1

Supplementary Material 2

Supplementary Material 3

Supplementary Material 4

## Data Availability

Data are available from the corresponding author upon reasonable request.

## References

[1] Peter SDSt, Juang D, Garey CL et al (2011) A novel measure for pectus excavatum: the correction index. J Pediatr Surg 46:2270–2273. 10.1016/j.jpedsurg.2011.09.009 22152863 10.1016/j.jpedsurg.2011.09.009

[2] Li H, Fan S, Kong X et al (2023) Academic performance in children with pectus excavatum: a real-world research with propensity score matching. Ther Adv Respir Dis 17:175346662311557. 10.1177/17534666231155779 10.1177/17534666231155779 PMC9972046 36846947

[3] Zens TJ, Berazaluce AMC, Jenkins TM et al (2022) The severity of pectus excavatum defect is associated with impaired cardiopulmonary function. Ann Thorac Surg 114:1015–1021. 10.1016/j.athoracsur.2021.07.051 34419435 10.1016/j.athoracsur.2021.07.051

[4] Ramadan S, Wilde J, Tabard-Fougère A et al (2021) Cardiopulmonary function in adolescent patients with pectus excavatum or carinatum. Bmj Open Respir Res 8:e001020. 10.1136/bmjresp-2021-001020 34326157 10.1136/bmjresp-2021-001020 PMC8323368

[5] Jaroszewski DE, Farina JM, Gotway MB et al (2022) Cardiopulmonary outcomes after the Nuss procedure in pectus excavatum. J Am Hear Assoc 11:e022149. 10.1161/jaha.121.022149 10.1161/jaha.121.022149 PMC9075480 35377159

[6] Wachtel FW, Ravitch MM, Grishman A (1956) The relation of pectus excavatum to heart disease. Am Heart J 52:121–137. 10.1016/0002-8703(56)90122-3 13326839 10.1016/0002-8703(56)90122-3

[7] Guller B, Hable K (1974) Cardiac findings in pectus excavatum in children: review and differential diagnosis. Chest 66:165–171. 10.1378/chest.66.2.165 4850886 10.1378/chest.66.2.165

[8] Ellis DG (1989) Chest wall deformities in children. Pediatr Ann 18:161–165. 10.3928/0090-4481-19890301-05 2664676 10.3928/0090-4481-19890301-05

[9] Raggio IM, Martínez-Ferro M, Bellía-Munzón G et al (2020) Diastolic and systolic cardiac dysfunction in pectus excavatum: relationship to exercise and malformation severity. Radiol Cardiothorac Imaging 2:e200011. 10.1148/ryct.2020200011 33778619 10.1148/ryct.2020200011 PMC7978017

[10] Saleh RS, Finn JP, Fenchel M et al (2010) Cardiovascular magnetic resonance in patients with pectus excavatum compared with normal controls. J Cardiov Magn Reson 12:73. 10.1186/1532-429x-12-73 10.1186/1532-429x-12-73 PMC3022801 21144053

[11] Töpper A, Polleichtner S, Zagrosek A et al (2016) Impact of surgical correction of pectus excavatum on cardiac function: insights on the right ventricle. A cardiovascular magnetic resonance study. Interact Cardiovasc Thorac Surg 22:38–46. 10.1093/icvts/ivv286 26487434 10.1093/icvts/ivv286

[12] Rodriguez-Granillo GA, Raggio IM, Deviggiano A et al (2019) Impact of pectus excavatum on cardiac morphology and function according to the site of maximum compression: effect of physical exertion and respiratory cycle. Eur Hear J - Cardiovasc Imaging 21:77–84. 10.1093/ehjci/jez061 10.1093/ehjci/jez061 30938414

[13] Nuss D, Kelly RE, Croitoru DP, Katz ME (1998) A 10-year review of a minimally invasive technique for the correction of pectus excavatum. J Pediatr Surg 33:545–552. 10.1016/s0022-3468(98)90314-1 9574749 10.1016/s0022-3468(98)90314-1

[14] Nuss D, Obermeyer RJ Jr REK (2016) Pectus excavatum from a pediatric surgeon’s perspective. Ann Cardiothorac Surg 5:493–500. 10.21037/acs.2016.06.04 27747183 10.21037/acs.2016.06.04 PMC5056929

[15] Haller JA, Kramer SS, Lietman SA (1987) Use of CT scans in selection of patients for pectusexcavatum surgery: a preliminary report. J Pediatr Surg 22:904–906. 10.1016/s0022-3468(87)80585-7 3681619 10.1016/s0022-3468(87)80585-7

[16] Tandon A, Wallihan D, Lubert AM, Taylor MD (2014) The effect of right ventricular compression on cardiac function in pediatric pectus excavatum. J Cardiovasc Magn Reson 16:P250. 10.1186/1532-429x-16-s1-p250 10.1186/1532-429x-16-s1-p250

[17] Fagelman KM, Methratta S, Cilley RE et al (2015) The Depression Index: an objective measure of the severity of pectus excavatum based on vertebral diameter, a morphometric correlate to patient size. J Pediatr Surg 50:1130–1133. 10.1016/j.jpedsurg.2014.11.043 25783321 10.1016/j.jpedsurg.2014.11.043

[18] Kim M, Lee K, Park H et al (2009) Development of new cardiac deformity indexes for pectus excavatum on computed tomography: feasibility for pre- and post-operative evaluation. Yonsei Med J 50:385–390. 10.3349/ymj.2009.50.3.385 19568601 10.3349/ymj.2009.50.3.385 PMC2703762

[19] Capunay C, Martinez-Ferro M, Carrascosa P et al (2020) Sternal torsion in pectus excavatum is related to cardiac compression and chest malformation indexes. J Pediatr Surg 55:619–624. 10.1016/j.jpedsurg.2019.05.008 31133283 10.1016/j.jpedsurg.2019.05.008

[20] Daunt SW, Cohen JH, Miller SF (2004) Age-related normal ranges for the Haller index in children. Pediatr Radiol 34:326–330. 10.1007/s00247-003-1116-1 14740200 10.1007/s00247-003-1116-1

[21] Robbers-Visser D, Boersma E, Helbing WA (2009) Normal biventricular function, volumes, and mass in children aged 8 to 17 years. J Magn Reson Imaging 29:552–559. 10.1002/jmri.21662 19243036 10.1002/jmri.21662

[22] Backhaus SJ, Schuster A, Lange T et al (2021) Impact of fully automated assessment on interstudy reproducibility of biventricular volumes and function in cardiac magnetic resonance imaging. Sci Rep 11:11648. 10.1038/s41598-021-90702-9 34078942 10.1038/s41598-021-90702-9 PMC8172876

[23] Krupickova S, Risch J, Gati S et al (2021) Cardiovascular magnetic resonance normal values in children for biventricular wall thickness and mass. J Cardiovasc Magn Reson 23:1. 10.1186/s12968-020-00692-2 33390185 10.1186/s12968-020-00692-2 PMC7780624

[24] Voges I, Caliebe A, Hinz S et al (2023) Pediatric cardiac magnetic resonance reference values for biventricular volumes derived from different contouring techniques. J Magn Reson Imaging 57:774–788. 10.1002/jmri.28299 35713958 10.1002/jmri.28299

[25] Backhaus SJ, Staab W, Steinmetz M et al (2019) Fully automated quantification of biventricular volumes and function in cardiovascular magnetic resonance: applicability to clinical routine settings. J Cardiovasc Magn Reson 21:24. 10.1186/s12968-019-0532-9 31023305 10.1186/s12968-019-0532-9 PMC8059518

[26] Ven JPG, Genuchten W, Sadighy Z et al (2023) Multivendor evaluation of automated MRI postprocessing of biventricular size and function for children with and without congenital heart defects. J Magn Reson Imaging 58:794–804. 10.1002/jmri.28568 36573004 10.1002/jmri.28568

[27] Wehrum T, Lodemann T, Hagenlocher P et al (2018) Age-related changes of right atrial morphology and inflow pattern assessed using 4D flow cardiovascular magnetic resonance: results of a population-based study. J Cardiovasc Magn Reson 20:38. 10.1186/s12968-018-0456-9 29898733 10.1186/s12968-018-0456-9 PMC6001162

[28] Dewhurst P, Coats L, Parikh JD et al (2020) The role of flow rotation in the adult right atrium: a 4D flow cardiovascular magnetic resonance study. Physiol Meas 41:035007. 10.1088/1361-6579/ab7d77 32143201 10.1088/1361-6579/ab7d77

[29] Truong VT, Li CY, Brown RL et al (2017) Occult RV systolic dysfunction detected by CMR derived RV circumferential strain in patients with pectus excavatum. PLoS ONE 12:e0189128. 10.1371/journal.pone.0189128 29228013 10.1371/journal.pone.0189128 PMC5724823

[30] Deviggiano A, Vallejos J, Vina N et al (2017) Exaggerated interventricular dependence among patients with pectus excavatum: combined assessment with cardiac MRI and chest CT. Am J Roentgenol 208:854–861. 10.2214/ajr.16.17296 28140622 10.2214/ajr.16.17296

