# Supplementary material for: Pectus excavatum: the effect of tricuspid valve compression on cardiac function
Source: Pediatr Radiol. 2024 Jul 9;54(9):1462–72. doi: 10.1007/s00247-024-05971-z (PMC11324711; doi:10.1007/s00247-024-05971-z)
Supplement: Supplementary file 1 — Supplementary Material 1 [file 247_2024_5971_MOESM1_ESM.docx]

**Pectus (Haller) Index (HI)**


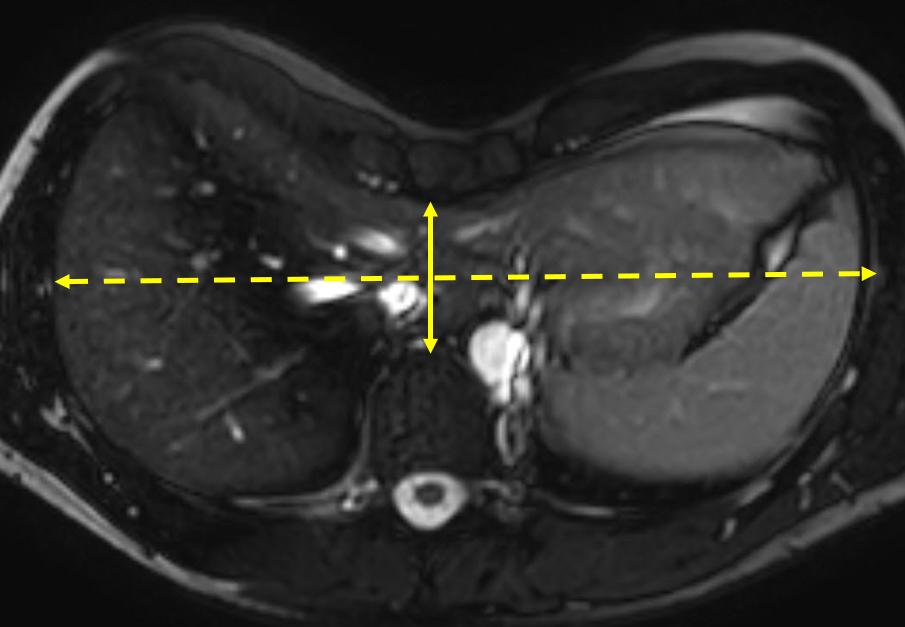


Fig. S3 Axial SSFP cine in a 17-year-old male with pectus excavatum and pectus index of 5.5. The pectus index (PE) is calculated by measuring the transverse diameter of the chest wall (dashed arrow) and dividing by distance between the spine and the anterior chest wall (solid arrow) at the lever where the anterior-posterior distance is least. A PE index above 3.25 is considered abnormal (1).

**Depression Index (DI)**


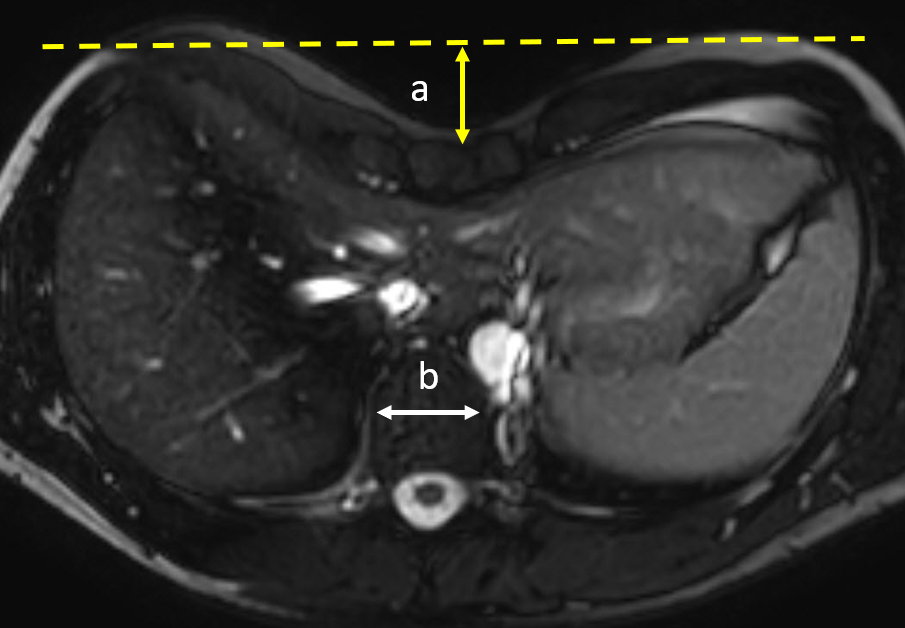


Fig. S4 Axial SSFP cine in a 17-year-old male with pectus excavatum and pectus index of 5.5. The depression index (DI) is calculated by placing a horizontal line along the most anterior displace bone or cartilage (dashed line) and dividing this distance by width of the vertebral body (solid arrow) at the same level as the pectus index was measured. DI = a/b. The reported threshold for DI to indicate a PE deformity severe enough to consider surgical correction is >0.2(2).

**Correction Index (CI)**


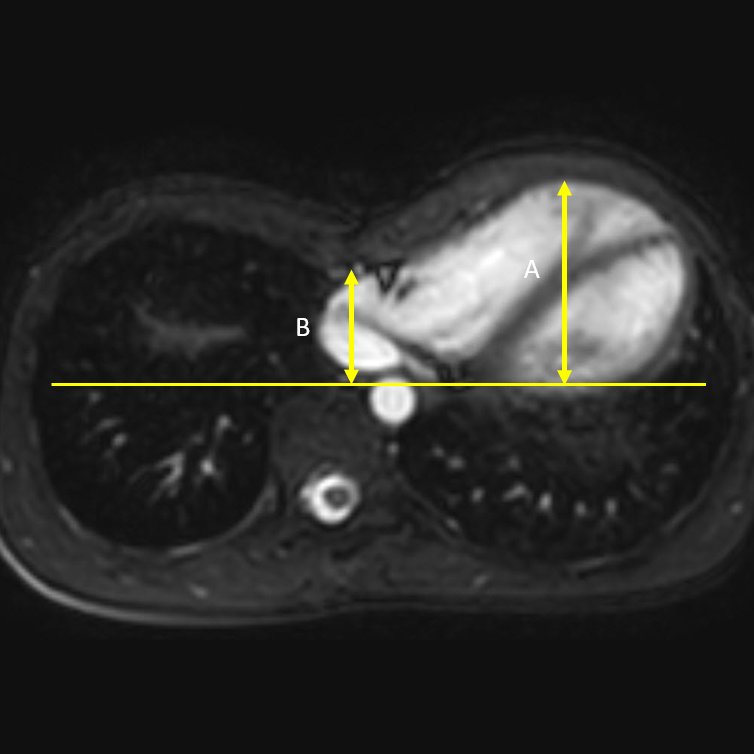


Fig. S5 Measuring the correction index (CI). Axial single shot SSFP with cardiac end systolic gating and respiratory gating in a 13-year-old male with pectus excavatum and pectus index of 6.7. The thin yellow line represents the anterior vertebral body at the site of narrowest anterior posterior diameter in the chest. The double arrowed line labeled “A” is the greatest transverse internal diameter of the chest at the same level as the narrowest portion of the chest represented by “B”. Correction index is {(A – B)/A}*100 %. (3)

**Modified Cardiac Compression Index (mCCI) and Modified Cardiac Asymmetry Index (mCAI)**


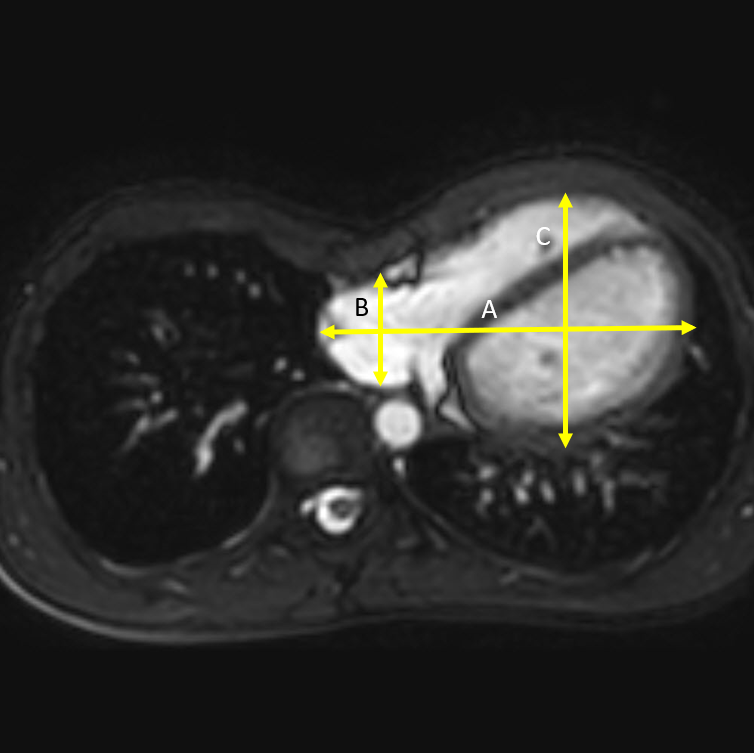


Fig. S6 Measuring the modified cardiac compression index (mCCI) and modified cardiac asymmetry index (mCAI). Axial single shot SSFP with cardiac end systolic gating and respiratory gating in a 13-year-old male with pectus excavatum and pectus index of 6.7 (same patient as Fig. S2). Select the first image above the inferior vena cava eustachian valve. This corresponds to approximately the mid TV. mCCI = A/B. mCAI = C/B.(4)

**Chest Wall Asymmetry Index (CWAI)**


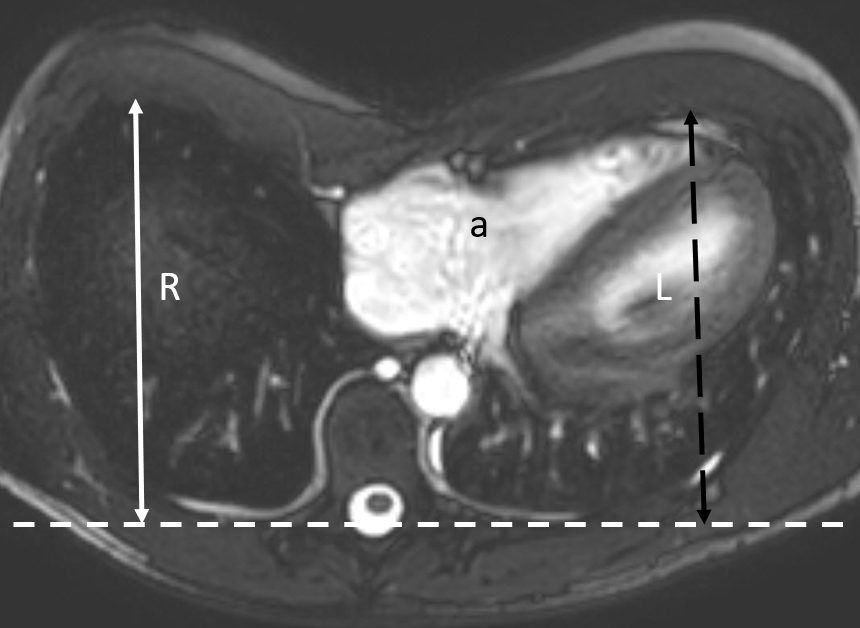


Fig. S7 Axial SSFP cine in a 17-year-old male with pectus excavatum and pectus index of 5.5. The same level of the cardiac compression index (mCCI) is where the chest wall asymmetry index is measured. We place a line that represents the posterior right and left chest cavity to account for the offset of the most aspect of the anterior chest cavity which is often not aligned with the posterior aspect of the chest cavity. The chest wall asymmetry index is calculated by dividing L by R.(4,5)

**Sternal Torsion**


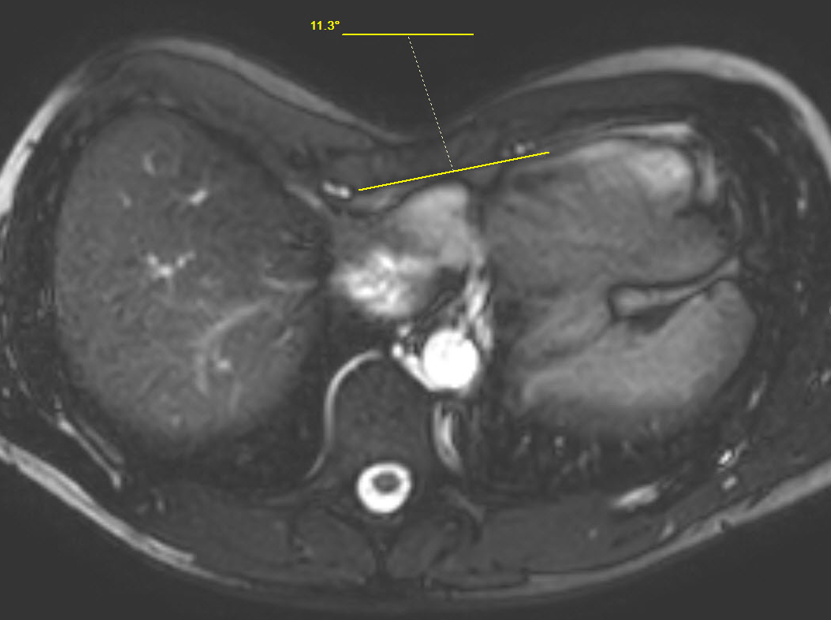


Fig. S8 Axial SSFP cine in a 17-year-old male with pectus excavatum and pectus index of 5.5. The sternal torsion(5) is measure above the xiphoid and costal chondral cartilages where the sternum can be clearly visualized. The Cobb angle tool is useful for making the measurement as shown above. In the report it is important to state whether the left side is down or the right side is down. Another convention is to report counterclockwise rotation as negative and clockwise rotation as positive. For example, this patient would be reported as: “Sternal angle -11 degrees with the right side down.”

1. Haller JA, Kramer SS, Lietman SA. Use of CT scans in selection of patients for pectusexcavatum surgery: A preliminary report. J Pediatr Surg. 1987;22(10):904–6.

2. Fagelman KM, Methratta S, Cilley RE, Wilson MZ, Hollenbeak CS. The Depression Index: an objective measure of the severity of pectus excavatum based on vertebral diameter, a morphometric correlate to patient size. J Pediatr Surg. 2015;50(7):1130–3.

3. Peter SDSt, Juang D, Garey CL, Laituri CA, Ostlie DJ, Sharp RJ, et al. A novel measure for pectus excavatum: the correction index. J Pediatr Surg. 2011;46(12):2270–3.

4. Kim M, Lee K, Park H, Kim HY, Kang EY, Oh Y, et al. Development of New Cardiac Deformity Indexes for Pectus Excavatum on Computed Tomography: Feasibility for Pre- and Post-Operative Evaluation. Yonsei Medical Journal. 2009;50(3):385–90.

5. Capunay C, Martinez-Ferro M, Carrascosa P, Bellia-Munzon G, Deviggiano A, Nazar M, et al. Sternal torsion in pectus excavatum is related to cardiac compression and chest malformation indexes. J Pediatr Surg. 2020;55(4):619–24.
