# Supplementary material for: Pectus excavatum: the effect of tricuspid valve compression on cardiac function
Source: Pediatr Radiol. 2024 Jul 9;54(9):1462–72. doi: 10.1007/s00247-024-05971-z (PMC11324711; doi:10.1007/s00247-024-05971-z)
Supplement: Supplementary file 4 — Supplementary Material 4 [file 247_2024_5971_MOESM4_ESM.docx]

**Cardiac MRI Pectus Excavatum protocol**

This is a generic list of sequences used for imagining the heart of pectus excavatum patients without specific sequence parameters. Despite the large number of sequences, these studies are performed in 30-40 minutes. This protocol has been used for years. We recently shortened the scheduling slots and now perform only sequences 1, 2, 5, 6, 7, 8, 10d and 11. This shortened protocol requires only 20 minutes.

1. Survey
2. Axial SSH BTFE non cine stack free breathing with cardiac end systolic gating and respiratory gating. Slice thickness 6 mm, Slice gap 8 mm, FOV 300-400 mm, Matrix 188 x 190
   1. This is used on all cardiac MRI in our laboratory. We have adopted it for measuring the pectus indices.
   2. There are other better choices for visualizing the sternum and measuring the indices which are easy to perform and quick such as mDixon, Single Shot FSE, etc. that will also serve this purpose.
3. Sagittal Stack nongated BTFE. Slice thickness 6 mm, Slice gap 6 mm, FOV 260 x 400 mm, Matrix 152 x 9
4. Coronal Stack nongated BTFE. Slice thickness 6 mm, Slice gap 6 mm, FOV 300-400 mm, Matrix 176 x 196
5. 2 chamber cine SSFP.
6. 4 chamber cine SSFP.
7. Short axis cine stack SSFP.
8. 3 chamber cine SSFP.
9. Axial cine stack SSFP from aortic arch through the heart.
10. Cine phase contrast
    1. Aortic valve
    2. Main pulmonary artery
    3. Atrioventricular valves
    4. “3-vessel” – an axial slice at the level of the right pulmonary artery which provides flow in the ascending aorta, superior venal cava, and descending aorta.
11. Coronal compressed sense 3d SSFP whole heart.
